# Parkinson’s disease in the United Arab Emirates (UAE): Emirates Neurology Society consensus guidelines

**DOI:** 10.3389/fneur.2026.1859775

**Published:** 2026-08-12

**Authors:** Shivam Om Mittal, Vittorio Iantorno, Ali Hassan, Pierre Krystkowiak, Pournamy Sarathchandran, Carl Johan Ramberg, Mohammad Alolama, Tanmoy Maiti, Mahesh Cirasanambati, Mazin Yousif Alsafi, Suhail Al Rukn

**Affiliations:** 1 Parkinson’s Disease and Movement Disorders Center, Neurological Institute, Cleveland Clinic Abu Dhabi, Abu Dhabi, United Arab Emirates; 2 King’s College Hospital London, Dubai, United Arab Emirates; 3 Tawam Hospital, Al Ain, United Arab Emirates; 4 Sh. Tahnoon Bin Mohammed Medical City, Al Ain, United Arab Emirates; 5 Specialized Rehabilitation Hospital, Abu Dhabi, United Arab Emirates; 6 University Hospital Sharjah, Sharjah, United Arab Emirates; 7 Al Qassimi Hospital Sharjah, Sharjah, United Arab Emirates; 8 Sheikh Khalifa Medical City Ajman, Ajman, United Arab Emirates; 9 Rashid Hospital, Dubai, United Arab Emirates; 10 Burjeel Medical City, Abu Dhabi, United Arab Emirates; 11 International University of Africa, Khartoum, Sudan

**Keywords:** device aided therapies, evidence based medicine (EBM), guidelines and recommendations, infusion pump therapies, multidisciplinary approach, neurodegenerating diseases, non-motor symptoms, United Arab Emirates

## Abstract

**Background:**

Parkinson’s disease (PD) incidence has risen by more than eightfold in the United Arab Emirates (UAE) between 1990 and 2019, representing one of the highest increases globally and creating substantial pressure on a fragmented, multi-payer health system. Region-specific guidance has been lacking, leading clinicians to extrapolate from international guidelines that do not fully address formulary heterogeneity, advanced therapy centralization, and cultural factors.

**Methods:**

Under the auspices of the Emirates Neurology Society, a national taskforce of 10 specialists representing major PD centers across the UAE used a three-round modified Delphi process informed by systematic literature review and key randomized controlled trials. Recommendations were graded using a GRADE-aligned system (A-C, Good Practice Points) and adapted to UAE formulary, service configuration, and cultural context.

**Results:**

Consensus was achieved on 146 recommendations across eight domains: (1) mandatory adoption of MDS-2015 diagnostic criteria; (2) age-stratified pharmacologic algorithms with levodopa as first-line for older or cognitively vulnerable patients; (3) structured non-motor symptom management; (4) rehabilitation and UAE cultural lifestyle adaptations including Ramadan fasting management, DBS considerations in women, family-centered care, and multilingual practice; (5) advanced therapy referral using the 5–2-1 rule integrated with the Making Informed Decisions to Aid Timely Management of Parkinson’s Disease (MANAGE-PD) tool, with five device-aided options (subcutaneous foslevodopa/foscarbidopa, apomorphine SC- subcutanous, LCIG- Levodopa Carbidopa Intestinal Gel, DBS- Deep Brain Stimulation, MRgFUS- MRI guided focussed ultrasound) presented through shared decision-making; (6) a three-tier service model; (7) UAE formulary mapping; and (8) center accreditation and registry metrics. Advanced therapies are centralized in accredited centers with clear minimum volume and multidisciplinary staffing criteria.

**Conclusion:**

These UAE consensus recommendations provide a pragmatic, context-adapted framework for PD management, emphasizing structured diagnosis, age-appropriate pharmacotherapy, systematic use of MANAGE-PD and 5-2-1 for advanced therapy referral, culturally sensitive practice adaptations, and a three-tier network model integrated through the Malaffi health information exchange and a planned national PD registry.

## 1 Introduction

The global prevalence of Parkinson’s disease (PD) has more than doubled since 1990, with projections suggesting over 12 million individuals will be affected by 2040 (1). In the Middle East and North Africa (MENA) region, and particularly in Gulf countries, age-standardized incidence and prevalence have risen faster than in most high-income settings; the United Arab Emirates (UAE) recorded an 854.71% increase in incidence between 1990 and 2019 (2). This surge coincides with population aging, substantial expatriate diversity, and rapid health system expansion (3).

The UAE healthcare system combines federal and emirate-level authorities, over 30 insurers, and variable formulary policies, resulting in unequal access to newer medications and advanced therapies across regions. While device-aided therapies (deep brain stimulation, magnetic resonance (MR)-guided focused ultrasound, levodopa-carbidopa intestinal gel (LCIG), continuous subcutaneous (SC) apomorphine infusion, and subcutaneous foslevodopa/foscarbidopa) are available, they are concentrated in a small number of centers (4). Existing international guidelines provide high-quality evidence syntheses but do not address UAE-specific issues such as formulary heterogeneity, centralized advanced therapy infrastructure, high consanguinity rates, Ramadan fasting, and multilingual family-centered care (5–9).

This article summarizes the UAE National PD Consensus Taskforce’s practical recommendations across eight domains, with particular attention to UAE-specific adaptations in pharmacotherapy, cultural practice, and service configuration. It is intended as a companion to the full UAE National PD Guidelines.

## 2 Methods

### 2.1 Taskforce composition and governance

The Emirates Neurology Society convened a national taskforce of 10 physicians actively managing PD in the UAE: 7 neurologists, 2 neurosurgeons, and 1 physical medicine and rehabilitation physician. Neurologists were required to have at least 5 years of PD practice in the UAE, with representation from Abu Dhabi, Dubai, and the Northern Emirates. Two neurologists were fellowship-trained in movement disorders. One neurosurgeon was fellowship-trained in stereotactic and functional neurosurgery (DBS) (4), and the second was certified in MR-guided focused ultrasound. The taskforce operated under Emirates Neurology Society governance.

### 2.2 Evidence review and consensus process

A systematic literature review (2015–2025) was performed across PubMed/MEDLINE, Embase, Cochrane Library, Web of Science, and guideline repositories. Included evidence comprised randomized controlled trials (RCTs), systematic reviews, observational cohort studies, and major international guidelines (5–8, 10). The taskforce used a three-round modified Delphi method with an 80% consensus threshold. Of 156 initial recommendations, 146 met the threshold and were finalized. Consensus agreement across all accepted recommendations met or exceeded the pre-defined 80% threshold. Three recommendations in the advanced therapy domain, specifically the relative sequencing of SC apomorphine versus LCIG, did not reach the threshold and were removed.

### 2.3 Evidence grading and UAE adaptation

Recommendations were graded as follows: Grade A (high-quality RCTs or meta-analyses), Grade B (moderate-quality RCTs or strong observational data), Grade C (lower-quality or indirect evidence), and Good Practice Points. UAE-specific adaptation addressed formulary availability, insurance authorization behavior, advanced therapy centralization, cultural considerations including Ramadan dosing (11) and family-centered decision-making (12), and integration with the Malaffi health information exchange.

## 3 Diagnostic criteria and baseline assessment

### 3.1 Mandatory use of MDS-2015 criteria

**Figure 1:**
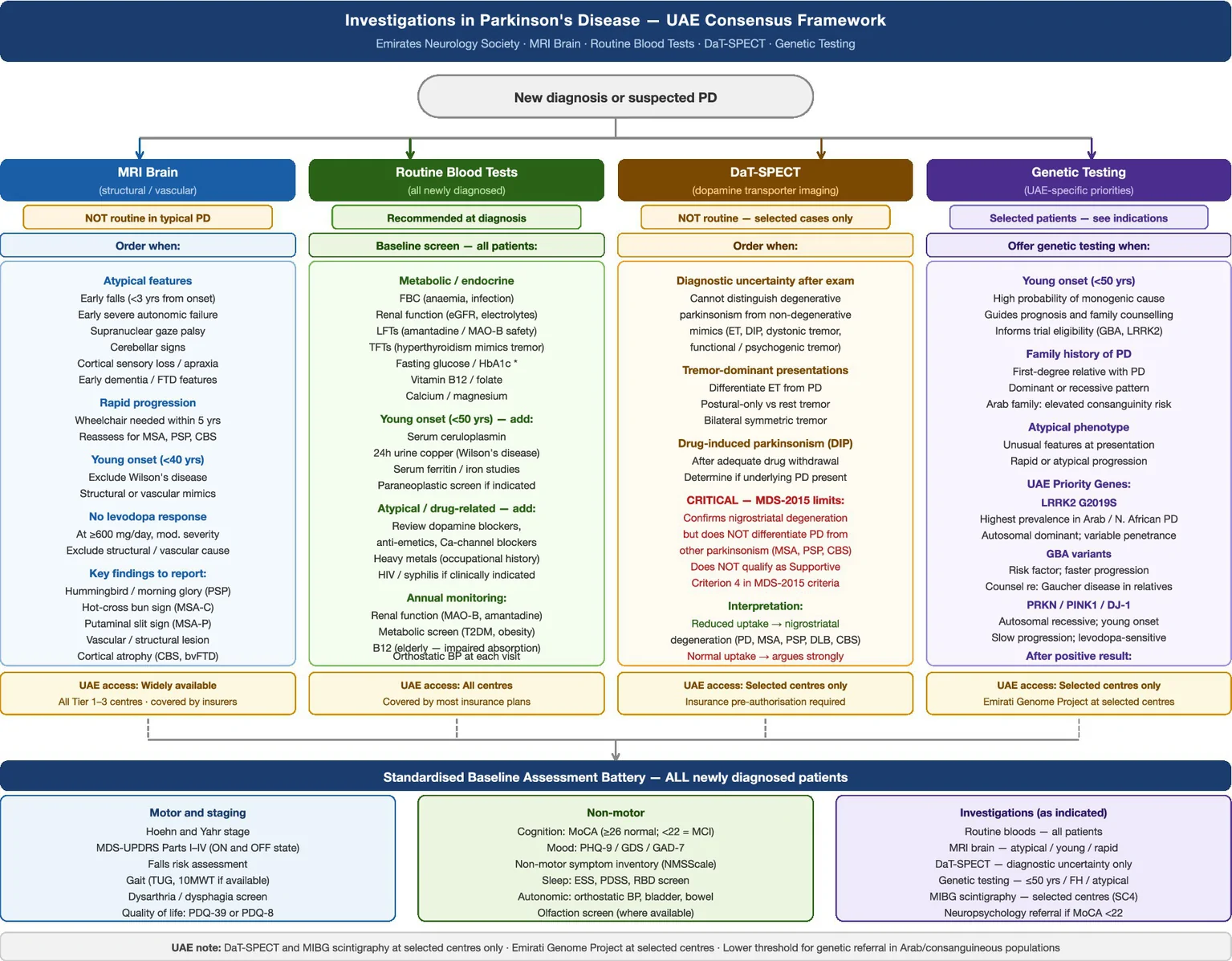
Clinical diagnostic pathway for Parkinson's disease (MDS-2015 criteria). The four-step MDS-2015 diagnostic process: Step 1 — confirm parkinsonism (bradykinesia plus rest tremor or rigidity); Step 2 — apply nine absolute exclusion criteria (any single criterion rules out PD); Step 3 — count red flags (>2 red flags renders probable PD undiagnosable); Step 4 — count supportive criteria (≥2 SC with zero RF = clinically established PD; RF counterbalanced by SC = clinically probable PD). DLB = dementia with Lewy bodies; MSA = multiple system atrophy; PSP = progressive supranuclear palsy; CBS = corticobasal syndrome; EC = exclusion criterion; RF = red flag; SC = supportive criterion. Emirates Neurology Society.

All suspected parkinsonism cases should be evaluated using the MDS 2015 Clinical Diagnostic Criteria for Parkinson’s disease, replacing older UK Brain Bank criteria in UAE practice. The MDS framework requires demonstration of parkinsonism (bradykinesia plus rest tremor or rigidity), evaluation of supportive criteria (clear levodopa response, levodopa-induced dyskinesia, rest tremor, and olfactory loss), identification of absolute exclusion criteria (9 items that individually rule out PD), and recognition of red flags (10 items requiring counterbalancing by supportive criteria). Adoption of the MDS-2015 criteria is a Grade A recommendation, supported by validation studies showing improved diagnostic accuracy and standardization. For the complete visual diagnostic pathway (see Figure 1; Table 1).

**Table 1:** Cardinal features of parkinsonism (prerequisite assessment before applying MDS-2015 criteria).

Cardinal feature	Definition	Assessment notes (MDS-UPDRS part 3)
BRADYKINESIA (required)	Slowness of movement AND progressive decrement in amplitude or speed (or progressive hesitations/halts) as movements are continued. Both elements must be present.	Finger tapping (3.4), hand movements (3.5), pronation-supination (3.6), toe tapping (3.7), foot tapping (3.8). A decline in either speed or amplitude on continuation is characteristic of PD.
REST TREMOR (at least one of two required)	4–6 Hz tremor in the fully resting limb, suppressed during movement initiation. Re-emergent tremor (reappears after brief cessation on sustained posture) qualifies if rest tremor was also observed.	Assess throughout examination (MDS-UPDRS 3.17, 3.18). Ensure limb is fully relaxed. Kinetic and postural tremors alone (MDS-UPDRS 3.15, 3.16) do NOT qualify for parkinsonism criteria.
RIGIDITY (at least one of two required)	Lead-pipe, velocity-independent resistance to passive movement of major joints with the patient in a relaxed position. Distinct from spasticity or paratonia.	Slow passive movement of major joints (MDS-UPDRS 3.3). Cogwheel phenomenon may be present but isolated cogwheeling WITHOUT lead-pipe rigidity does NOT fulfill minimum requirements.

### 3.2 Investigations and genetic testing

Brain MRI is recommended selectively for atypical presentations, rapid progression, absent levodopa response, or early-onset disease (<40 years). Dopamine transporter imaging (DaT-SPECT) is reserved for cases where differentiation between degenerative parkinsonism and non-degenerative mimics (essential tremor, drug-induced parkinsonism, and functional tremor) remains unclear after full clinical examination or when the MDS parkinsonism clinical criteria are not fulfilled. There is a critical caveat: DaT-SPECT does NOT differentiate PD from other parkinsonian syndromes, such as multiple system atrophy (MSA), progressive supranuclear palsy (PSP), and corticobasal syndrome (CBS) and does not qualify as MDS-2015 Supportive Criterion 4. Both DaT-SPECT and MIBG scintigraphy are available at selected centers only; referral should be made via specialist pathways with insurance pre-authorization. FDG-PET CT brain may be considered in diagnostically challenging cases where atypical parkinsonian syndromes (PSP, MSA, and CBS) cannot be excluded on clinical grounds alone. Characteristic hypometabolism patterns—midbrain and striatal in PSP, cerebellar in MSA-C, and asymmetric frontoparietal in CBS—can provide supportive evidence when clinical and MRI findings remain equivocal. FDG-PET CT is not recommended as a routine investigation for typical PD; availability is limited to selected Tier 2/3 centers in the UAE and insurance pre-authorization is required (Table 2).

**Table 2:** Baseline assessment battery for all newly diagnosed patients with Parkinson’s disease.

Domain	Assessment tools	Purpose
Motor/staging	Hoehn & Yahr stage; MDS-UPDRS Parts I–IV (ON and OFF state); TUG; 10MWT; PDQ-39 or PDQ-8	Severity staging; treatment decisions; quality of life baseline; registry entry
Cognition	MoCA (≥26 normal; <22 = MCI threshold); full neuropsychological assessment if MoCA <22	Guides dopamine agonist safety; DBS candidacy; driving assessment
Mood/psychiatric	PHQ-9 (depression); GAD-7 (anxiety); GDS in older patients	High prevalence; undertreated; significant driver of quality of life
Non-motor screen	NMS Scale/NMSQ; ESS + PDSS (sleep); SCOPA-AUT (autonomic)	Comprehensive non-motor burden; identifies treatment targets
Autonomic	Lying/standing BP at every visit; bladder and bowel symptom review; post-void residual if indicated	Identifies orthostatic hypotension, urinary and GI dysfunction
Falls/rehabilitation	TUG; Berg Balance Scale; dysphagia screen (UPDRS 2.3); swallowing questionnaire	Identifies falls risk; guides physiotherapy and SLT referral
Investigations	As per Figure 2 (selected per clinical indication)	Excludes secondary causes; informs genetic counseling; supports diagnosis

PDQ-39 or PDQ-8 should be repeated annually to track quality-of-life trajectory. Document all baseline assessments in a structured manner in the patient record to enable longitudinal tracking and registry reporting.

**Figure 2:**
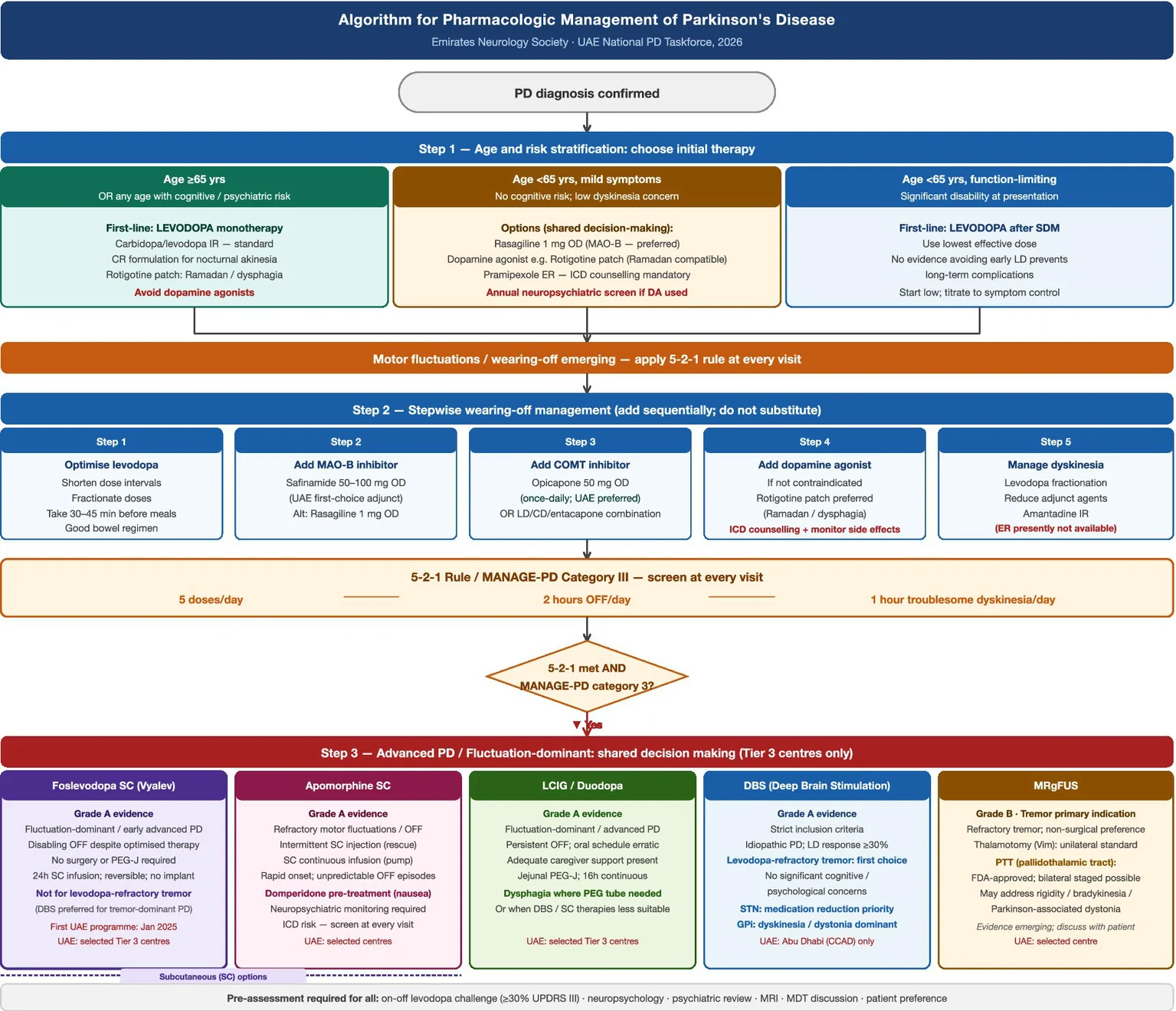
Clinical investigation and genetic screening workflow for parkinsonism. Four-column framework: (1) MRI brain — indications, expected findings, and UAE access; (2) routine blood tests — baseline battery for all newly diagnosed patients; (3) DaT-SPECT — selective indications, interpretation caveats (does NOT differentiate PD from MSA/PSP/CBS and does NOT qualify as MDS-2015 Supportive Criterion 4); (4) genetic testing — priority genes by population (LRRK2 G2019S in Arab/North African PD; GBA as risk modifier; PRKN/PINK1/DJ-1 for young-onset recessive PD). Evidence grades A–B per MDS EBM review. Emirates Neurology Society.

Genetic testing is recommended for young-onset cases (<=50 years), strong family history, or atypical phenotypes, with particular attention to LRRK2, GBA, PRKN, PINK1, and DJ-1. The high prevalence of LRRK2 G2019S in Arab and North African PD populations, combined with elevated consanguinity rates in the UAE, justifies a lower threshold for genetic referral than European guidelines suggest. Genetic counseling is mandatory post-result. The Emirati Genome Project provides genetic testing at selected centers in the UAE, and clinicians are encouraged to refer through this pathway where available. For a summary of investigation indications (see Figure 2).

### 3.3 Baseline staging and assessment battery

At diagnosis, all patients should undergo a standardized baseline assessment: Hoehn & Yahr stage; MDS-UPDRS Parts I-IV (ON and OFF state); MoCA (> = 26 normal; 21–25 = MCI; <21 = dementia) (13, 14); PHQ-9 and GAD-7; Non-Motor Symptoms Scale (NMSScale), which systematically captures non-motor burden including autonomic dysfunction, sleep, mood, and cognition; autonomic review (lying/standing BP; bowel and bladder); sleep questionnaires (ESS, PDSS); quality of life (PDQ-39 or PDQ-8); and falls risk assessment (TUG, Berg Balance Scale) (15). This structured battery supports prognostication, treatment planning, registry entry, and longitudinal quality-of-life tracking (Table 3).

**Table 3:** Non-motor symptom screening and management in Parkinson's disease.

Domain	Screening tool	Management approach	Grade	When to refer
Cognitive impairment/dementia	MoCA; neuropsychology if MoCA <22	Rivastigmine (first-line for PD dementia). Review anticholinergic burden. Avoid antipsychotics except quetiapine/clozapine (specialist). Annual MoCA.	A	MoCA <22; suspected dementia; driving concerns
Depression/anxiety	PHQ-9; GAD-7; GDS (older patients)	SSRIs/SNRIs first-line. Avoid TCAs (anticholinergic, arrhythmic risk). Do not defer to psychiatry before initiating first-line treatment in PD.	A	Severe or treatment-resistant symptoms
Psychosis/hallucinations	Clinical review each visit	1. Exclude delirium/infection/metabolic. 2. Reduce dopaminergic load stepwise. 3. Quetiapine 12.5–25 mg nocte or pimavanserin (not available in UAE). 4. Clozapine (Tier 3 only). NEVER use haloperidol, risperidone, olanzapine.	B	Any new psychosis — Tier 2/3 urgently
Sleep — insomnia/EDS	ESS; PDSS; sleep diary	Sleep hygiene; adjust dopaminergic timing; melatonin. Modafinil for EDS if no contraindication.	B	Suspected sleep apnoea; severe EDS; RBD with injury risk
REM sleep behavior disorder	History (patient + bed partner)	Low-dose clonazepam (0.25–0.5 mg) or melatonin (3–12 mg) at bedtime. Bed safety measures. Counsel: RBD is a marker of synucleinopathy.	B	Injury risk; diagnostic uncertainty
Orthostatic hypotension	Lying/standing BP at every visit (3 min standing)	Non-pharm first: fluids 2–2.5 L/day, compression stockings, head elevation. Fludrocortisone or midodrine if persistent.	B	Severe or syncopal episodes; cardiologist input
Constipation	Bowel frequency at each visit	Hydration; fiber; mobility; macrogol first-line. Avoid anticholinergics. Review medication contributors.	GPP	Fecal impaction; obstruction signs
Urinary dysfunction	Symptom review; post-void residual if indicated	Bladder diary; behavioral strategies. Mirabegron preferred choice. Solifenacin with caution (cognitive risk). Urologist for retention or structural cause.	B	Retention; refractory urgency; haematuria
Sialorrhea/drooling	UPDRS item 2.2	Posture and swallowing advice (SLT). Botulinum toxin (Tier 2/3). Glycopyrrolate if botulinum toxin unavailable.	B	Moderate-severe; aspiration risk
Pain	Type characterization at each visit	Optimize dopaminergic therapy (dystonic pain). Physiotherapy (musculoskeletal). Analgesics matched to pain phenotype.	GPP	Neuropathic or central pain refractory to optimization
Fatigue	Systematic enquiry	Treat contributing factors: sleep disorder, depression, anemia, hypothyroidism, dehydration. Optimize dopaminergic regimen. Exercise.	GPP	Refractory; consider specialist review
Dysphagia	UPDRS 2.3; swallowing questionnaire	SLT referral. VFSS if moderate-severe. Modified diet/texture as indicated. PEG discussion in advanced disease.	B	Aspiration pneumonia risk; weight loss; choking episodes

Seppi et al. (7).

## 4 Pharmacologic management and formulary-adapted sequencing

### 4.1 Levodopa as the gold-standard therapy

Levodopa (with carbidopa or benserazide) remains the most effective symptomatic therapy across disease stages and is recommended as first-line for patients > = 65 years or any age with cognitive vulnerability (5, 10, 16). Landmark RCTs (PD MED and ELLDOPA) demonstrate superior motor benefit and quality of life compared with dopamine agonists or MAO-B inhibitors as initial therapy (10). Motor complications (wearing-off and dyskinesia) are better interpreted as disease-duration related than as evidence of levodopa toxicity; there is no robust evidence that delaying levodopa initiation prevents long-term complications (5, 16, 17). Immediate-release carbidopa/levodopa is the default formulation; controlled-release preparations address nocturnal or early-morning akinesia, acknowledging lower bioavailability.

### 4.2 Age-stratified initial therapy

- Age > = 65 years, or any age with cognitive or psychiatric risk: levodopa monotherapy should be used as first-line treatment. The patient should avoid dopamine agonists due to the significantly higher risk of hallucinations, impulse-control disorders, excessive daytime somnolence, and falls in this group (5, 10).
- Age <65 years, mild symptoms, low dyskinesia concern: MAO-B inhibitor (rasagiline 1 mg) OD preferred (consensus choice: once-daily dosing, established UAE formulary availability, and a favorable evidence base for motor benefit; safinamide is preferred as Step 2 adjunct for wearing-off given its dual MAO-B + Na^+^ channel mechanism) or dopamine agonist may be considered as treatment. Selegiline is at present not available in the UAE. Rotigotine patches are preferred when an agonist is chosen (Ramadan-compatible; no oral timing). Full ICD counseling is mandatory. Annual neuropsychiatric monitoring is advised (5).
- Age <65 years, function-limiting symptoms: levodopa is appropriate as first-line treatment after shared decision-making, prioritizing function and quality of life over theoretical dyskinesia delay (5, 10).

### 4.3 Managing motor fluctuations—a stepwise/multi-layered strategy

**Figure 3:**
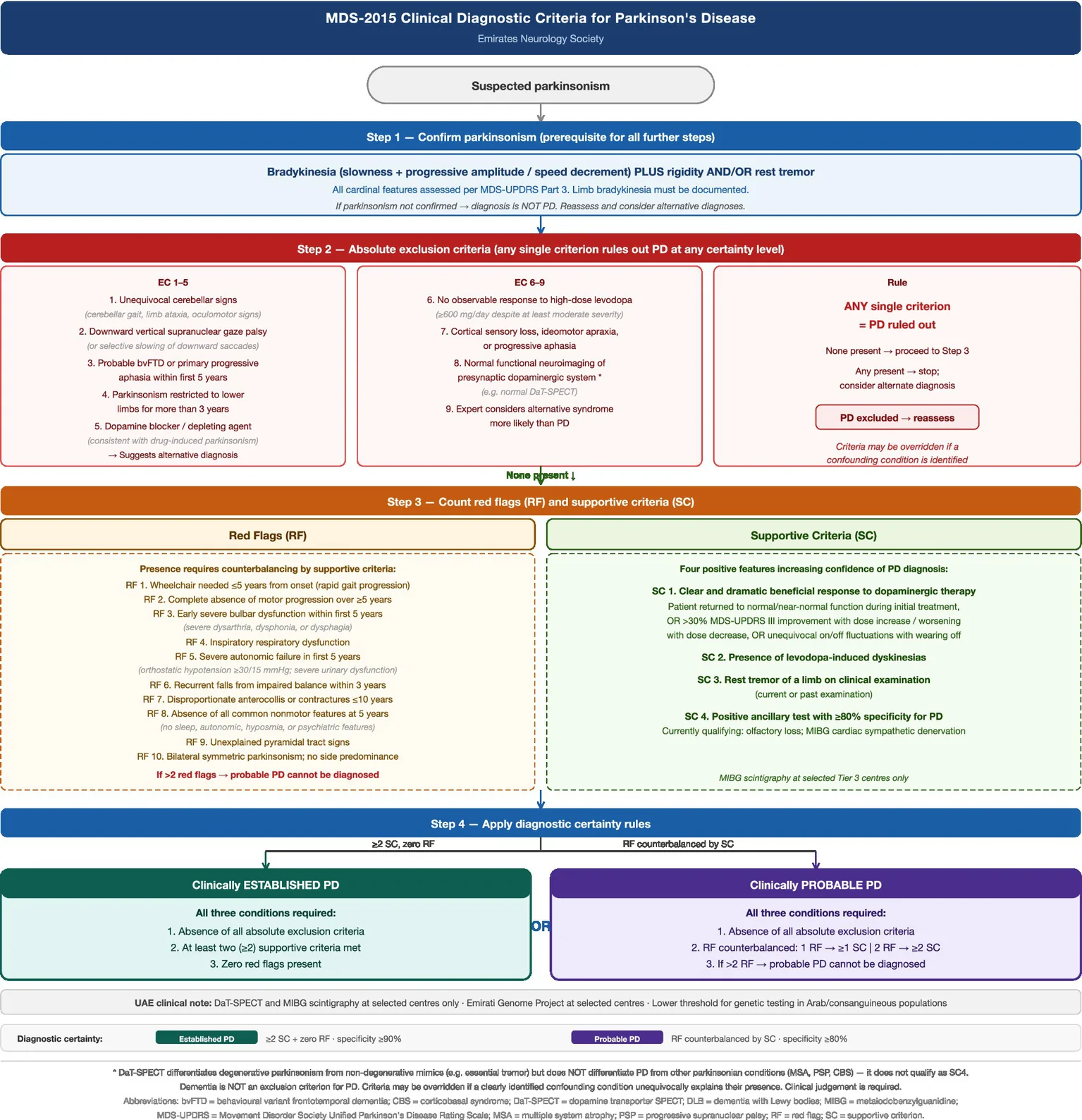
Algorithm for pharmacologic management and therapeutic sequencing in Parkinson’s disease, adapted for UAE clinical practice. Three-phase algorithm: Phase 1 — age- and risk-stratified initial therapy (levodopa monotherapy for age ≥65 or cognitive risk; MAO-B inhibitor or dopamine agonist for age <65 mild; levodopa for function-limiting symptoms at any age); Phase 2 — five-step wearing-off management (optimize levodopa → add MAO-B inhibitor → add COMT inhibitor [opicapone or Stalevo; stand-alone entacapone not routinely available in UAE] → add/optimize dopamine agonist → manage dyskinesia with amantadine IR [ER not available UAE]); Phase 3 — advanced/device-aided therapy when 5-2-1 criteria met and MANAGE-PD suboptimal/poor. UAE shared decision-making for five device-aided options: (1) Foslevodopa SC/Vyalev — preferred first option, least invasive, selected Tier 3 centers; (2) Apomorphine SC — rescue injection or continuous pump, domperidone pre-treatment; (3) LCIG/Duodopa — 16h jejunal infusion via PEG-J; (4) DBS — first choice for levodopa-refractory tremor or if pump declined, Abu Dhabi (CCAD); (5) MRgFUS — tremor primary indication, PTT bilateral staged possible, selected center. LD = levodopa; DA = dopamine agonist; MAO-B = monoamine oxidase B inhibitor; COMT-I = catechol-*O*-methyltransferase inhibitor; ICD = impulse control disorder; SDM = shared decision-making; DBS = deep brain stimulation; CCAD = Cleveland Clinic Abu Dhabi. Sources: de Bie RMA et al. (6); Mittal et al. (11); Mittal SO et al. (4).

Wearing off typically emerges within 3–5 years of levodopa initiation. The taskforce recommends the following sequential approach, adding steps as needed rather than replacing earlier interventions (5):

- Step 1: Optimize levodopa: inter-dose intervals should be shortened into fractionated doses. These should be taken 45–60 min before meals. Constipation should be treated actively (as this impairs absorption) (5).
- Step 2: Add MAO-B inhibitor: about 50–100 mg of safinamide once daily (OD) is preferred (dual mechanism: MAO-B inhibition + Na + channel blockade—this reduces both OFF time and dyskinesia). Rasagiline 1 mg OD is an alternative (5).
- Step 3: Add COMT inhibitor: opicapone 50 mg OD is preferred in the UAE—it has the better adherence. Alternatively, fixed combination Stalevo can be used. Stand-alone entacapone is NOT routinely available in the UAE—use opicapone or Stalevo (5).
- Step 4: Add/optimize dopamine agonist: a dopamine agonist should be added or optimized if not contraindicated by age or cognitive/psychiatric profile. Rotigotine patches are preferred (continuous delivery; Ramadan-compatible). ICD counseling and annual neuropsychiatric screen is mandatory (5).
- Step 5: Manage dyskinesia: amantadine IR should be used for troublesome levodopa-induced dyskinesia. Levodopa fractionation should be used. Adjunct dopaminergic agents should be reduced where feasible. Note: amantadine ER is not currently available in the UAE (5).
- Step 6: Advanced therapy: when disabling fluctuations persist despite fully optimized oral/patch regimens, the 5-2-1 rule + Making Informed Decisions to Aid Timely Management of Parkinson’s Disease (MANAGE-PD) tool should be applied and Tier 3 used promptly (18–20). The terminology “advanced Parkinson’s disease” carries a psychological burden that may delay device-aided therapy referral—patients who remain functionally active often reject referral when they hear “advanced.” The recently proposed concept of Fluctuation-Dominant Parkinson’s disease (FD-PD) reframes this transition as a therapeutic opportunity: FD-PD describes patients in whom motor and non-motor fluctuations are the predominant disease experience and whose primary unmet need is inadequate dopaminergic coverage, decoupling eligibility from overall disease burden and naming the intermediate disease phase where most patients live (21). For the complete pharmacologic management algorithm (see Figure 3).

### 4.4 UAE formulary—essential medicines (March 2026)

The taskforce considers the following agents essential and expects availability across UAE formularies: multiple strengths of levodopa/carbidopa (IR and CR), levodopa/benserazide, rotigotine patch, pramipexole IR/ER, rasagiline, safinamide, opicapone, Stalevo, amantadine IR, rivastigmine, SSRIs/SNRIs, quetiapine, and clozapine (with REMS monitoring at specialist centers) (5, 6). Streamlined prior authorization is strongly recommended for safinamide, opicapone, and all advanced infusion therapies. There is limited availability of ropinirole ER at present.

## 5 Non-motor symptoms—screening and management

Non-motor symptoms affect virtually all patients with PD and are the principal driver of disability, caregiver burden, hospitalization, and quality of life (6, 15). They are systematically under-recognized in routine consultations. A brief structured review is recommended at every meaningful visit: the review should cover mood, sleep, cognition, constipation, dizziness on standing, hallucinations, urinary symptoms, pain, fatigue, swallowing, and drooling (6).

### 5.1 Cognitive impairment and dementia

The MoCA screening should take place at every significant visit. Rivastigmine patch or capsule is first-line therapy for PD dementia (6). The anticholinergic burden should be reviewed. Antipsychotics should be avoided, except for quetiapine or clozapine (specialist). Annual MoCA should be carried out. Referral should be made if MoCA <21 or if driving is a concern.

### 5.2 Depression and anxiety

Screening should take place using PHQ-9 and GAD-7. SSRIs/SNRIs are first-line treatment (caution: concomitant use with rasagiline or safinamide is not recommended in standard prescribing; some healthcare insurers in the UAE consider this a contraindication—discuss risk–benefit individually); tricyclics should be avoided (anticholinergic and arrhythmic risk) (6). Referrals to psychiatry should not delay starting the first line treatment). Referral should be made for severe or treatment-resistant symptoms.

### 5.3 Psychosis and hallucinations

The management sequence is as follows (6): (1) exclude delirium, infection, metabolic disturbance, and medication toxicity; (2) reduce dopaminergic load stepwise (reduce levodopa dose as a last resort); (3) use quetiapine 12.5–25 mg nocte (or pimavanserin if available); (4) use clozapine (Tier 3 only, REMS monitoring). The following should never be used in PD: haloperidol, risperidone, or olanzapine. They carry high risk of severe, irreversible parkinsonism worsening.

### 5.4 Sleep disorders

Screening should take place with ESS and PDSS. Sleep hygiene should be managed first; dopaminergic timing should be adjusted; and you can give melatonin for REM sleep behavior disorder (RBD) (3–12 mg nocte) or low-dose clonazepam (0.25–0.5 mg) (6). The patient and bed partner should be counseled on injury risk from RBD. RBD is a prodromal marker of synucleinopathy. Referral should be made if sleep apnoea or severe EDS are suspected.

### 5.5 Orthostatic hypotension

Lying/standing BP should be measured at every visit (3 min standing). Non-pharmacologic intervention should be prioritized: fluids 2–2.5 L/day, compression stockings, and head elevation. Fludrocortisone or midodrine can be used if persistent (6). Referral should be made for syncopal episodes or cardiologist input.

### 5.6 Constipation

Bowel frequency should be assessed at every visit and, relatedly, hydration, fiber, and mobility should be monitored. Macrogol is a first-line pharmacologic agent. Anticholinergics should be avoided. Medication contributors should be reviewed (opioids and anticholinergics). Aggressive management of constipation is also important as a strategy to optimize levodopa absorption (6).

### 5.7 Sialorrhea/drooling

Posture and swallowing advice should be followed (SLT). Botulinum toxin injection into the parotid and/or submandibular glands at Tier 2/3 is an effective and evidence-based intervention (22). Glycopyrrolate should be used if botulinum toxin is unavailable. Referral should be made if there is moderate–severe or aspiration risk.

### 5.8 Dysphagia

Dysphagia should be assessed at every visit (MDS-UPDRS 2.3; swallowing questionnaire). SLT referral should be made. Videofluoroscopic swallowing study (VFSS) can be used if condition is moderate to severe. Modified diet/texture should be followed as indicated. Discuss the possibility of percutaneous endoscopic gastrostomy (PEG) in cases of advanced disease. Dysphagia increases aspiration pneumonia risk—a leading cause of death in advanced PD (6).

### 5.9 Pain and fatigue

The pain type should be characterized at each visit (dystonic pain responds to dopaminergic optimization; musculoskeletal pain to physiotherapy; neuropathic/central pain to specialist review). Fatigue should be handled as follows: treat contributing factors (sleep disorder, depression, anemia, hypothyroidism, and dehydration) and optimize the dopaminergic regimen (6). Exercise remains the broadest evidence-based intervention for fatigue in PD.

## 6 Rehabilitation, lifestyle, and UAE cultural adaptations

Rehabilitation is a core therapeutic modality in PD—not an optional adjunct. Exercise has demonstrated neuroprotective and disease-modifying potential in animal models and consistent clinical benefits for motor function, balance, falls reduction, mood, and quality of life in human trials (15). Allied health referrals should be made as actively as medication changes. The taskforce recommends prescribing 150–300 min per week of moderate aerobic activity combined with resistance training for all patients with PD, with physiotherapy referral (ideally LSVT BIG certified) initiated at diagnosis or at first sign of motor decline (Table 4).

**Table 4:** Rehabilitation and allied health referral framework for Parkinson's disease.

Intervention	Refer when	Key components	Grade	UAE note
Physiotherapy (LSVT BIG)	All patients at diagnosis; any motor decline	150–300 min/week moderate aerobic + resistance training. Gait, balance, fall prevention. Dual-task training. Treadmill and cycling beneficial.	A	LSVT BIG available at Tier 2/3 centers
Speech-language therapy (LSVT LOUD)	Hypophonia; dysarthria; any dysphagia	Voice amplification and articulation training. Swallowing safety; VFSS if moderate-severe dysphagia.	A	Available at Tier 2/3 centers; home visits for Northern Emirates
Occupational therapy	Functional decline; home safety concern; driving assessment needed	ADL training, home adaptations, fatigue management, assistive devices, driving assessment.	B	Home assessment visits; telemedicine available
Exercise program	At diagnosis for all; community class options	Aerobic, resistance, balance, stretching. Boxing (Rock Steady), cycling, dance — all demonstrated benefit in PD trials. Droxidopa is currently not availble in UAE.	A	Morning/evening scheduling in UAE summer; air-conditioned facilities preferred
Falls prevention program	Any falls history; Hoehn & Yahr ≥3; freezing of gait	Multifactorial falls assessment. Dual-task training. Home hazard removal. Hip protectors. Carer education.	A	High priority given UAE home caregiving model
Dietitian/clinical nutrition	Weight loss; dysphagia; constipation; T2DM	Protein distribution advice (large protein meals impair levodopa absorption). Texture modification. Hydration optimization.	GPP	Key Ramadan counseling role
Neuropsychology	MoCA <22; behavioral symptoms; driving cessation	Cognitive rehabilitation. Carer and family education. Support for lifestyle adjustment decisions.	B	Driving cessation counseling important in UAE context
Social work/care coordination	All patients; advanced disease priority; family support needs	Care planning. Carer support and respite. Financial assessment. Advance care planning. Telemedicine access (Northern Emirates).	GPP	Culturally adapted support essential; nursing homes culturally stigmatized

### 6.1 UAE cultural and lifestyle adaptations

#### 6.1.1 Ramadan fasting and Parkinson’s disease

Ramadan is often observed by Muslim patients for 1 lunar month each year, during which they abstain from food, drink, and oral medication between dawn (Fajr) and sunset (Maghrib). The fasting window varies from approximately 11 to 18 h depending on season and geographic latitude. For patients with PD, this creates a significant pharmacological challenge: levodopa has a short half-life of 60–90 min, and medication gaps exceeding 4 h carry a material risk of akinetic crisis, aspiration, falls, dysphagia, and dehydration. Despite Islamic religious texts permitting individuals with chronic illness to break the fast (with Fidya as an alternative), many patients elect to fast, often without disclosing this to their neurologist (11, 23).

Key recommendations (11, 23, 24) are as follows:

- Rotigotine transdermal patch is the first-choice dopaminergic agent during Ramadan. It provides continuous 24-h dopaminergic delivery without oral intake, eliminating the fasting-window medication gap. It should be applied to non-sun-exposed skin and the site rotated daily. Individuals should be monitored for hyperdosing in UAE summer heat (increased transdermal absorption at temperatures up to 45–50 degrees C) (11).
- Oral levodopa adjustment: Doses can be consolidated into the non-fasting window (Iftar to Suhoor). Extended-release formulations should be considered. Levodopa should never be stopped abruptly as there is a risk of akinetic crisis and a neuroleptic malignant syndrome-like state. Levodopa gaps exceeding 4 h should be avoided at any stage (11, 23).
- Once-daily agents at Iftar: Rasagiline 1 mg OD or opicapone 50 mg OD can be taken at Iftar without disrupting timing. MAO-B inhibitors extend the effective duration of levodopa and reduce OFF time during the fasting window (11).
- Motor fluctuations: Patients who meet the 5–2-1 should be strongly advised not to fast or to seek religious guidance on the use of Fidya. Prolonged medication gaps in fluctuating patients carry high risk of akinetic crisis requiring emergency admission (11).
- Gradual dose adjustment: The dopaminergic dose can be reduced by 10–15% before Ramadan begins (to reduce dyskinesia risk from consolidated dosing at Iftar/Suhoor) and restored gradually after Ramadan ends (11).
- Protein-levodopa interaction: Oral levodopa can be taken 45–60 min before eating at Iftar. Heavy protein meals compete with levodopa absorption (large neutral amino acid competition) and may worsen motor control in the hours after breaking the fast (11).
- Hydration and constipation: Dehydration worsens constipation, which impairs levodopa absorption. Fluid intake should be maximized during non-fasting hours (minimum 2 L). Constipation should be treated aggressively before and during Ramadan (11, 23).
- Pre-Ramadan review: All patients with PD who plan to fast should be reviewed at least 2–4 weeks before Ramadan begins. Rotigotine patches should be initiated or titrated in advance. Patients and families should be counseled on warning signs of prolonged OFF, aspiration, and when to break the fast and seek emergency care (11).

#### 6.1.2 DBS surgery in women—cultural and practical considerations

Deep brain stimulation surgery is equally effective in women with PD. However, several cultural and practical considerations are relevant in the UAE and broader Arab Muslim context and should be addressed proactively during the DBS evaluation process (4).

- No head shaving required: at Cleveland Clinic Abu Dhabi (CCAD), minimally invasive stereotactic techniques allow DBS lead placement through small incisions behind the hairline without requiring head shaving. This should be explicitly communicated during counseling (4, 25).
- IPG placement: The internal pulse generator (IPG) is placed subcutaneously in the infraclavicular region. A lower chest or abdominal wall placement may be preferable in some women for comfort and concealment under traditional clothing (e.g., abaya and thobe). Preference should be documented pre-operatively (4).
- Gender-concordant care: Where possible, women should be offered the option of a same-gender neurologist for DBS programming and follow-up and a same-gender nurse specialist for device education. A chaperone should be offered during all physical examinations—this is standard practice under UAE healthcare regulations (4).

#### 6.1.3 Family-centered care

In Arab and South Asian cultures prevalent in the UAE, family members play a central role in health decision-making, caregiving, and treatment adherence. Decisions about advanced therapies—DBS, infusion treatments, and care home placement—are often made collectively by the extended family, with significant influence from senior men in the family. This cultural norm should be acknowledged and incorporated into the clinical encounter (12).

- Include family at key decision points: Advanced therapy counseling (DBS, LCIG, and foslevodopa), disclosure of diagnosis, and advance care planning discussions should explicitly include family members. Family education sessions should be offered at diagnosis and at each major treatment transition in both Arabic and English (12).
- Home caregiving preference: Institutionalization and care home placement are culturally stigmatized across most Arab populations. This should be acknowledged explicitly in care plans; MDT support should be structured accordingly (home visits for physiotherapy, OT, and speech therapy; telemedicine for Northern Emirates) (12).
- Stigma reduction: Social stigma associated with PD as a chronic visible neurological illness has been documented across MENASA countries and may cause delayed diagnosis and delayed presentation for advanced therapies. Proactive education, bilingual patient information materials, and community awareness programs are recommended (12).

#### 6.1.4 Heat, climate, and outdoor activity

The UAE summer presents specific management challenges for patients with PD:

- Rotigotine patch absorption: Transdermal drug absorption increases significantly with skin temperature and humidity. Patients should be monitored for signs of rotigotine overdosing in the summer months: dyskinesia, nausea, postural hypotension, and somnolence. Patches should be applied in air-conditioned rooms, and direct sun on the patch site should be avoided.
- Heat-related dehydration: Patients with PD are particularly vulnerable given compromised autonomic regulation, constipation, and reduced thirst sensation. Dehydration worsens constipation, reduces levodopa absorption, and increases orthostatic hypotension risk. The minimum fluid intake target is 2 L/day, increasing to 2.5 L in the summer months.
- Outdoor exercise timing: Patients should be advised to exercise before 8 a.m. or after 6 p.m., or in air-conditioned environments, during the summer. Exercise remains essential—heat should not be an excuse for inactivity but requires appropriate scheduling.

#### 6.1.5 Driving safety

Driving is critically important for independence in the UAE, where public transport infrastructure varies significantly between emirates. Several PD-related factors impair driving safety: motor fluctuations (sudden OFF episodes), excessive daytime somnolence (especially with dopamine agonists), cognitive impairment, and visual dysfunction. Clinicians have a medicolegal obligation to assess and document driving safety at each visit.

- When to assess? Assessment should take place when the following symptoms are observed: motor fluctuations with unpredictable OFF episodes; daytime somnolence or sleep attacks (mandatory discussion before initiating any dopamine agonist); MoCA <24; freezing of gait; or postural instability. UAE Road and Transport Authority (RTA) regulations should be applied when assessing fitness to drive. Referral to occupational therapy for formal driving assessment should take place where indicated.
- Sleep attacks—medicolegal requirement: All patients commenced on a dopamine agonist must be explicitly counseled about the risk of sudden-onset sleep (sleep attacks) and advised not to drive until tolerance to this effect is established. The counseling conversation should be documented in the medical record (6).

#### 6.1.6 Sexual dysfunction—an underreported non-motor symptom in the UAE

Sexual dysfunction is a common non-motor symptom in PD affecting both men and women, but it is significantly under-recognized and underreported in Middle Eastern populations due to religious, cultural, and social barriers.

Clinicians should specifically and sensitively enquire about sexual function at follow-up visits, using gender-appropriate questioning and offering same-gender clinical team presence. Screening should use NMSS item 12 as an entry point. Erectile dysfunction in men may signal autonomic failure (MDS-2015 Red Flag RF5). Impulse control disorder (hypersexuality) must be actively screened in all patients on dopamine agonists (12).

#### 6.1.7 Multilingual and multicultural patient population

The UAE population comprises over 200 nationalities, with Arabic, English, Hindi, Urdu, Tagalog, and Malayalam among the most prevalent languages in PD-relevant age groups. The majority of the population are expatriates. This presents specific challenges for informed consent, patient education, medication adherence counseling, and advance care planning (9, 12).

Patient education materials should be provided in Arabic and English as a minimum standard; where patient populations are large, Hindi and Urdu versions should also be considered. Professional medical interpreters (not family members) should be used for all treatment decision conversations, advanced therapy counseling, and formal consent procedures. Medical translation apps are insufficient for complex neurological counseling (9, 12).

## 7 Advanced therapies, MANAGE-PD, and the 5-2-1 rule

### 7.1 Identifying candidates—the 5-2-1 rule

The 5-2-1 rule provides a simple clinic-friendly bedside screen for patients who may be inadequately controlled through current therapeutic interventions and should be considered for advanced therapy evaluation. It should be applied at every outpatient visit for patients with established PD and its criteria are as follows (18):

- > = 5 levodopa doses per day: This causes an excessive dosing burden; poor compliance is likely; and the drug–food interaction risk is high (18).
- > = 2 h of OFF time per day: This creates a significant OFF burden despite oral therapy; there may be some functional impairment (18).
- > = 1 h of troublesome dyskinesia per day: There is persistent ON-state dyskinesia, limiting function or quality of life (18).

Any patient meeting one or more criteria warrants MANAGE-PD assessment. Patients who meet the 5–2-1 criteria AND who are classified as suboptimally or poorly controlled on MANAGE-PD should be referred promptly to a Tier 3 PD Centre for advanced therapy evaluation (18, 19).

### 7.2 MANAGE-PD assessment tool

MANAGE-PD (Making Informed Decisions to Aid Timely Management of Parkinson’s Disease) is a validated, free, online and paper-based tool designed to help physicians identify patients with PD who are not well controlled on oral medications. It is a brief structured questionnaire completed by the clinician in the outpatient setting to categorize patients as well-controlled, suboptimally controlled, or poorly controlled on current oral/patch therapy (18). It standardizes the trigger for advanced therapy evaluation across all levels of care. Annual MANAGE-PD screening is recommended for all patients with established motor complications across Tier 1 and Tier 2 (18). The tool should be embedded in electronic medical records and clinical templates.

### 7.3 Advanced therapy options—UAE availability and selection

Advanced therapies should only be initiated in accredited Tier 3 centers after standardized pre-assessment: on–off levodopa challenge (≥30% MDS-UPDRS III improvement required), formal neuropsychological testing, psychiatric review, high-resolution MRI, and multidisciplinary team discussion. Insurance pre-authorization should be initiated concurrently with the referral—do not wait for authorization before referring (19).

Therapy selection must be guided by shared decision-making. The clinician presents the benefits, limitations, and practical demands of each option; the patient chooses based on individual preference, lifestyle, caregiver support, and clinical profile. Five device-aided options are available in the UAE, presented below in order from least to most invasive. The complete treatment algorithm is presented in Figure 3.

#### 7.3.1 Foslevodopa/foscarbidopa subcutaneous infusion (Vyalev)—preferred first option

Continuous subcutaneous foslevodopa/foscarbidopa (Vyalev) is the recommended first device-aided option for fluctuation-dominant and early advanced PD. It is the least invasive of the five options: no surgery, no PEG-J, reversible, and administered as a 24-h subcutaneous infusion via abdominal site rotation. It provides stable, continuous dopaminergic stimulation without the need for a surgical implant. It is not appropriate for levodopa-refractory tremor, where DBS remains the treatment of choice. Subcutaneous foslevodopa/foscarbidopa programs are available at selected accredited Tier 3 centers in the UAE (26).

#### 7.3.2 Apomorphine subcutaneous (SC)

Apomorphine is a potent dopamine agonist with a rapid onset of action (within 5–10 min of injection), making it the fastest-acting rescue therapy available for PD. It can be used in two ways: (1) intermittent subcutaneous injection for unpredictable OFF episodes, where it provides rapid rescue; or (2) continuous subcutaneous apomorphine infusion (CSAI) via a portable pump worn for 12–16 h per day, providing sustained motor control in patients with frequent or prolonged OFF periods. CSAI reduces OFF time by approximately 50–60% and can decrease troublesome dyskinesias (27, 28). It is non-surgical, reversible, and can usually be initiated in an outpatient setting. Daily pump management—including setup, site rotation, and pump handling—is required. Clinicians should counsel patients and caregivers that long-term observational data report a 35–60% discontinuation rate within 1–2 years, primarily due to neuropsychiatric side effects (hallucinations), skin nodules at infusion sites, and waning efficacy (28, 29). Domperidone pre-treatment is mandatory before initiation to prevent nausea and vomiting and must be continued during titration. Neuropsychiatric monitoring and ICD screening are required at every visit. Available at selected accredited centers in the UAE (27, 28).

#### 7.3.3 Levodopa-carbidopa intestinal gel (LCIG/duodopa)

LCIG delivers levodopa/carbidopa gel directly into the proximal jejunum via a percutaneous endoscopic gastrojejunostomy (PEG-J) tube and a portable pump, providing continuous 16-h intestinal infusion. By bypassing the stomach and delivering the drug at a constant rate, it eliminates the variable gastric emptying that contributes to wearing-off in advanced PD. LCIG is indicated for patients with persistent motor fluctuations and troublesome OFF periods despite optimized oral therapy and is particularly suited to patients with dysphagia where a PEG tube is independently clinically indicated, or where DBS and subcutaneous infusion therapies are less suitable. It requires PEG-J placement under endoscopic or radiological guidance, committed caregiver involvement for daily pump management, and regular nursing support. Available at selected accredited Tier 3 centers in the UAE (20).

#### 7.3.4 Deep brain stimulation (DBS)

The DBS is a surgical procedure in which thin electrodes are implanted stereotactically into specific deep brain targets—most commonly the subthalamic nucleus (STN) or the globus pallidus internus (GPi)—and connected via subcutaneous extensions to a rechargeable pulse generator (IPG) implanted in the chest wall. Continuous electrical stimulation of these targets modulates the abnormal neural circuits responsible for PD motor symptoms. DBS effectively alleviates tremor, rigidity, bradykinesia, motor fluctuations, and dyskinesia and significantly improves quality of life (19). The procedure is performed under local or general anesthesia and typically requires a 1-to-3-day hospital stay for lead implantation, with a separate day-case procedure for IPG implantation. Programming is performed in the outpatient setting.

STN-DBS is favored when medication reduction is a priority and cognitive and mood profiles are reassuring; it can achieve 50–60% reduction in OFF time and equivalent reduction in dyskinesia. GPi-DBS is preferred when dyskinesia or dystonia is prominent, or when mood stability is a concern, as it has a more favorable neuropsychiatric side-effect profile. DBS is the first choice for levodopa-refractory tremor, or when a patient declines pump-based therapy. Key eligibility criteria include idiopathic PD with a clear levodopa response (≥30% MDS-UPDRS III improvement); absence of dementia; absence of uncontrolled psychiatric illness; and adequate social support for device management. Patients and families should be counseled about DBS early in the disease course—at the point of first fluctuations—to allow informed planning. Common misconceptions include the requirement for head shaving: at CCAD (Cleveland Clinic Abu Dhabi, currently the only center performing DBS in the UAE), minimally invasive stereotactic techniques allow lead placement through small incisions behind the hairline without complete head shaving (4). Perioperative PD medication management requires careful planning to avoid akinetic crisis (30).

#### 7.3.5 MR-guided focused ultrasound (MRgFUS)

MRgFUS is a incisionless, non-implant procedure that uses precisely focused ultrasound waves, guided by real-time MRI, to create a small therapeutic lesion in a targeted brain structure. No incision, no implant, and no anesthesia are required. In standard practice, Vim thalamotomy provides unilateral tremor control and remains the established MRgFUS approach for tremor-dominant PD. Evolving evidence supports pallidothalamic tractotomy (PTT) as an FDA-approved target that can be performed bilaterally in staged sessions, with potential benefits for rigidity, bradykinesia, and Parkinson-associated dystonia beyond tremor; this evidence is still emerging and patient selection should be individualized at an experienced center. MRgFUS is suitable for patients who are not DBS candidates due to surgical preference, comorbidity, or access considerations, and who have predominantly unilateral or tremor-dominant disease. It is not suitable for management of OFF time, complex dyskinesia, or multi-domain bilateral motor disability in the standard approach. It is available at selected accredited centers in the UAE.

## 8 Three-tier service model, registry, and implementation

### 8.1 Tiered care pathway

The recommended care network comprises a hub-and-spoke networked model, adapted from evidence-based frameworks including Dutch ParkinsonNet, Lombardy’s regional PD network, and Canadian specialist-care outcome data, which collectively demonstrate reduced fractures, hospitalizations, costs, and long-term care placement under coordinated specialist-network care (31–33):

- Tier 1: General Neurology comprises MDS-2015 diagnosis; first-line therapy initiation; stable disease follow-up; simple non-motor management; annual MANAGE-PD screening (18). Referral triggers are as follows: 5–2-1 criteria are met; MANAGE-PD suboptimal/poor; atypical features; young onset; advanced therapy consideration; diagnostic uncertainty.
- Tier 2: Parkinson’s Clinics comprises complex pharmacotherapy; structured non-motor management; rehabilitation coordination; systematic 5–2-1 and MANAGE-PD (18); advanced therapy candidate identification; genetic counseling. Referral triggers are as follows: advanced therapy candidacy confirmed; failed Tier 2 optimization; atypical parkinsonism; DBS/LCIG/MRgFUS/infusion evaluation.
- Tier 3: PD Centres of Excellence comprises DBS, LCIG, foslevodopa/foscarbidopa programs (4, 19); atypical parkinsonism management; genetic counseling; research; education; national PD registry coordination.

### 8.2 Referral triggers—tier 1 to tier 2/3

Refer to Tier 2 when the following is present: diagnostic uncertainty or atypical features; first motor fluctuations or dyskinesia emerging; MANAGE-PD suboptimal/poor (18); complex non-motor symptoms (psychosis, dementia, refractory depression, autonomic failure); young onset PD (<=50 years); significant side effects requiring regimen restructuring; rehabilitation coordination; or second opinion requested.

Refer to Tier 3 when the following is present: 5-2-1 criteria are met + MANAGE-PD poor/suboptimal control (18, 19); advanced therapy candidacy confirmed or under evaluation; atypical parkinsonism (MSA, PSP, CBS, and DLB); genetic counseling (LRRK2, GBA positive result); diagnostic uncertainty after Tier 2 assessment; research or national PD registry enrolment; pre-Ramadan medication planning in complex cases (11).

### 8.3 Quality indicators and implementation

Quality indicators tracked include the following: proportion of new diagnoses documented using MDS-2015 criteria; time from 5-2-1 trigger to advanced therapy referral (18); 12-month continuation rates for infusion therapies (20); and registry-based outcomes (OFF-time reduction, MDS-UPDRS improvement, PDQ-39, and falls rates). The Malaffi health information exchange underpins cross-provider data sharing in Abu Dhabi and will be expanded across emirates. A national PD registry is planned for launch in 2027, with participation a core expectation for accredited centers (3).

Key implementation priorities are as follows: standardized MDS-2015 documentation templates and MANAGE-PD should be embedded in electronic health records; implementation of standardized insurance authorization pathways for advanced therapies; bilingual (Arabic/English) patient education materials should be provided at diagnosis (9, 12); telemedicine capacity should be built for Northern Emirates; there should be an annual guideline review with a 3-year formal update.

## 9 Conclusion

The UAE National PD Consensus Taskforce provides a structured, regionally adapted framework for PD care that combines international evidence (5, 6, 10, 15) with UAE-specific realities (3, 4, 11). Central features include mandatory use of MDS-2015 diagnostic criteria, age-stratified levodopa-anchored pharmacotherapy (5, 10), systematic use of MANAGE-PD and 5–2-1 criteria to avoid delayed advanced therapy referral (18, 19), explicit recognition of UAE formulary constraints (opicapone and Stalevo; no stand-alone entacapone; rotigotine patch preferred for Ramadan and dysphagia), a comprehensive program of culturally sensitive adaptations (11, 12, 23–25, 27), and a three-tier network model integrated via Malaffi and a national PD registry (31–33). These recommendations aim to reduce practice variation, improve equitable access to advanced therapies, and position the UAE as a regional leader in evidence-based PD care.
